# Machine learning-based identification of colorectal advanced adenoma using clinical and laboratory data: a phase I exploratory study in accordance with updated World Endoscopy Organization guidelines for noninvasive colorectal cancer screening tests

**DOI:** 10.3389/fonc.2024.1325514

**Published:** 2024-02-23

**Authors:** Huijie Wang, Xu Cao, Ping Meng, Caihua Zheng, Jinli Liu, Yong Liu, Tianpeng Zhang, Xiaofang Li, Xiaoyang Shi, Xiaoxing Sun, Teng Zhang, Haiying Zuo, Zhichao Wang, Xin Fu, Huan Li, Huanwei Zheng

**Affiliations:** ^1^ Department of Endoscopy, Shijiazhuang Traditional Chinese Medicine Hospital, Shijiazhuang, China; ^2^ Department of Gastroenterology, Shijiazhuang Traditional Chinese Medicine Hospital, Shijiazhuang, China; ^3^ Department of Anus & Intestine Surgery, Shijiazhuang Traditional Chinese Medicine Hospital, Shijiazhuang, China; ^4^ Institute of Traditional Chinese Medicine, North China University of Science and Technology, Tangshan, China; ^5^ Graduate School, Hebei North University, Zhangjiakou, China; ^6^ Research and Development Department, Wuhan Metware Biotechnology Co., Ltd, Wuhan, China

**Keywords:** advanced colorectal adenoma, machine learning, non-invasive test, risk assessment, adjustable thresholds

## Abstract

**Objective:**

The recent World Endoscopy Organization (WEO) guidelines now recognize precursor lesions of colorectal cancer (CRC) as legitimate screening targets. However, an optimal screening method for detecting advanced adenoma (AA), a significant precursor lesion, remains elusive.

**Methods:**

We employed five machine learning methods, using clinical and laboratory data, to develop and validate a diagnostic model for identifying patients with AA (569 AAs vs. 3228 controls with normal colonoscopy). The best-performing model was selected based on sensitivity and specificity assessments. Its performance in recognizing adenoma-carcinoma sequence was evaluated in line with guidelines, and adjustable thresholds were established. For comparison, the Fecal Occult Blood Test (FOBT) was also selected.

**Results:**

The XGBoost model demonstrated superior performance in identifying AA, with a sensitivity of 70.8% and a specificity of 83.4%. It successfully detected 42.7% of non-advanced adenoma (NAA) and 80.1% of CRC. The model-transformed risk assessment scale provided diagnostic performance at different positivity thresholds. Compared to FOBT, the XGBoost model better identified AA and NAA, however, was less effective in CRC.

**Conclusion:**

The XGBoost model, compared to FOBT, offers improved accuracy in identifying AA patients. While it may not meet the recommendations of some organizations, it provides value for individuals who are unable to use FOBT for various reasons.

## 1 Introduction

Colorectal cancer (CRC) represents a significant threat to residents of China, contributing substantially to the societal burden (1). In China, CRC-related new cases and deaths account for 9.87% and 8.01% of all malignant tumor incidence and mortality, respectively (2). Addressing this public health challenge effectively is of paramount importance (3).

The goal of CRC screening is to reduce mortality and morbidity by identifying treatable CRC cases and precursor lesions, while minimizing health risks and individual burdens (4). In 2023, the CRC Screening Committee of the World Endoscopy Organization (WEO) issued guidelines for evaluating novel non-invasive screening tests for CRC. These guidelines recommend a dual-step screening process, starting with a non-invasive test and, if positive, followed by a colonoscopy. The non-invasive test should be capable of identifying individuals with an increased likelihood of CRC or advanced precursor lesions (5). Advanced adenoma (AA), an important precursor lesion, is currently considered to carry a significantly elevated risk (6). Although the fecal immunochemical test (FIT) is currently the most widely used non-invasive test, its sensitivity for early detection of CRC, especially AA, remains suboptimal (7). As a result, there is an urgent need for more accurate and non-invasive screening strategies that can identify AA, thereby improving survival rates among CRC patients (8).

Recent clinical guidelines from the Asian Pacific Gastroenterology and Digestive Endoscopy highlight the superiority of combining biomarkers over single biomarkers for detecting colorectal neoplasia (8). Machine learning, based on feature combinations, has emerged as a powerful and effective method for predictive analytics. Its successful application in diagnosis, prediction, and treatment selection has received considerable recognition (9). Routine medical laboratory tests are widely used in China and have become an essential part of modern healthcare (10). The results of these tests may contain more information than even the most experienced clinician can discern, making them suitable for analysis through artificial intelligence to uncover subtle interrelationships (11).

Predictive and diagnostic models based on routine clinical and laboratory data have been developed for various cancers (12–15). However, they often exhibit low sensitivity for AAs due to their non-specific symptoms and distinct risk factors compared to those of CRC (16).

The CRC Screening Committee of the WEO outlines a four-phase evaluation process for new tests, starting with Phase I studies involving limited cohorts or case-control studies (5). Based on this premise, we conducted a Phase I exploratory case-control study using clinical and laboratory data. The aim is to construct a machine-learning diagnostic model for identifying AA and to assess its ability to meet the objectives of non-invasive screening tests, as outlined in the WEO guidelines. These objectives include diagnostic performance regarding the adenoma-carcinoma sequence, adjustable thresholds of positivity, and comparison with validated non-invasive screening tests.

## 2 Materials and methods

### 2.1 Study design and population

**Figure 1:**
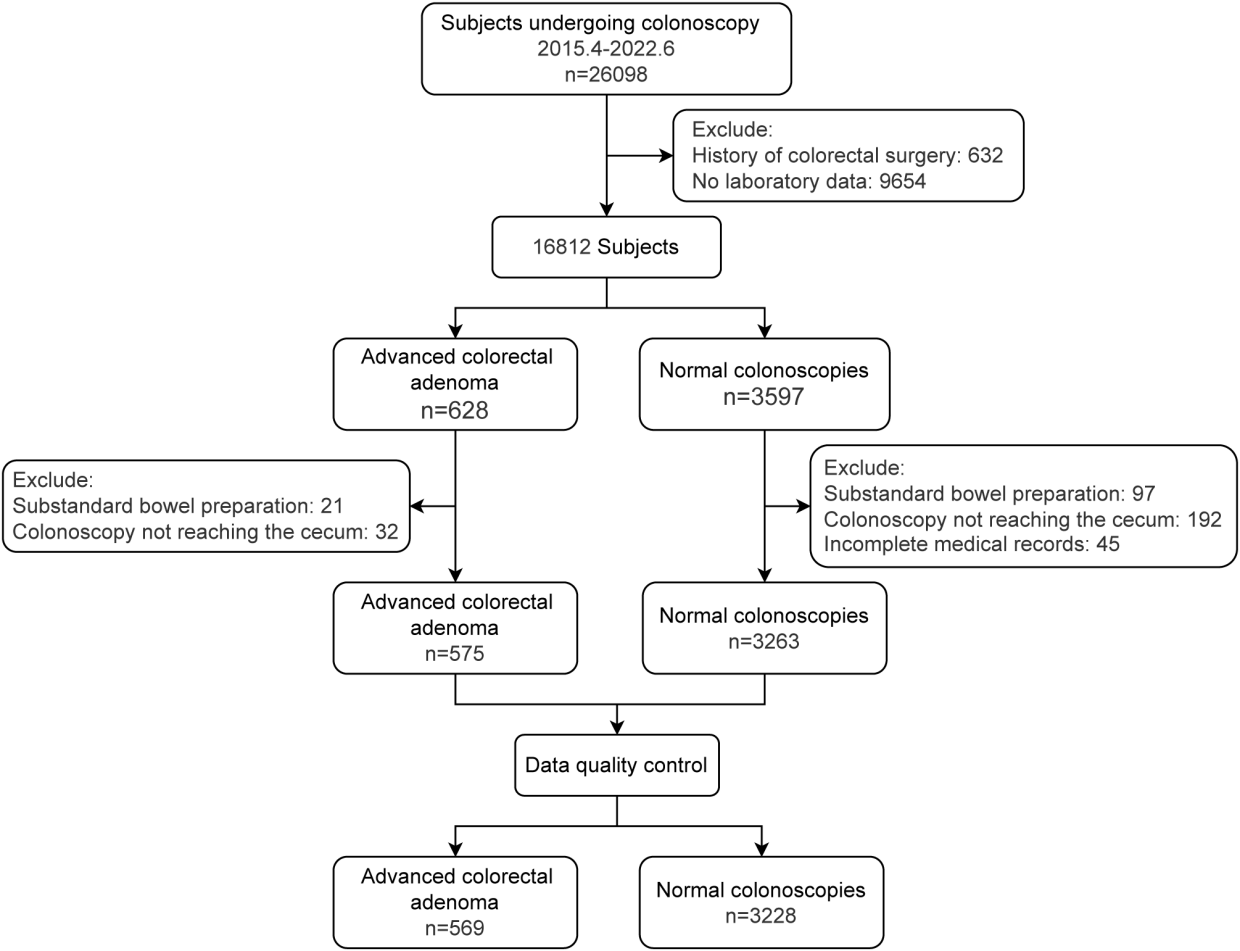
Study population flowchart.

We conducted a retrospective case-control study, comprising a case group with AA (17) and a control group with normal colonoscopies. The objective was to develop (train) and validate (test) a model for diagnosing AA. AA is defined as an adenoma that exhibits any of the following characteristics: size ≥ 1 cm, presence of tubulovillous or villous components, or high-grade dysplasia. The model was constructed based on features obtained as part of routine clinical care, which included demographic characteristics, lifestyle factors, and clinical features (including comorbidities and laboratory indicators). We ensured that data from at least one laboratory test were available within one month before the colonoscopy. In addition, we validated the outcome model in other populations including non-advanced adenoma (NAA) and CRC. NAA refers to an adenoma that does not meet the definition of AA. All subjects were identified using colonoscopy and pathohistological diagnoses obtained from medical records between April 2015 and June 2022. The exclusion criteria were as follows: a history of colorectal surgery, incomplete medical records, substandard bowel preparation, a colonoscopy that did not reach the cecum, and cases that did not meet the standards of data quality control (QC) (Detail for **Figure 1**). Specifically, the exclusion criteria for CRC did not include substandard bowel preparation and whether the colonoscope reached the cecum.

The retrospective study received approval from the Ethics Committee of Shijiazhuang Traditional Chinese Medicine Hospital (NO.20220919029), and the requirements for informed consent were waived due to the retrospective nature of the study.

### 2.2 Data collection

The present study collected demographic characteristics (age, sex, and marital status), smoking and drinking information (including never, former, and current usage), comorbid conditions, and laboratory test results (routine blood and urine tests, fecal occult blood test [FOBT], biochemistry, tumor markers, and coagulation function). Notably, qualitative FOBT is more prevalent in Chinese hospitals than quantitative FIT. The FOBT method employed in this study was immunocolloid gold (the FOBT was considered positive when the hemoglobin level was greater than or equal to 0.2 μg/ml diluent).

Detailed test methods for laboratory data included in the machine learning analyses were presented in **Supplementary Table 1**.

### 2.3 Feature screening

During the data QC process, we retained the features of interest and excluded features with a missing rate exceeding 20% or samples with a missing rate exceeding 50%. For continuous variables with missing values in features retained after QC, we imputed them using k-nearest neighbors (KNN, K = 15). Missing values in categorical variables with a missing rate of less than 20% were imputed using grouped plurality while missing rates of 20% or more were imputed with the new phenotype “MISS”.

Subsequently, we initially screened categorical and continuous variables using the chi-square test and random forest, respectively, to eliminate features with minimal or no impact on grouping. The remaining features’ importance was ranked using the logistic regression (LR), random forest (RF), and least absolute shrinkage and selection operator (LASSO) methods. This step was repeated 10 times to mitigate random bias in data splitting. We took the intersection of the features selected by different models and weighted the importance of each feature, summing them to obtain the importance of weighted features for manual screening.

### 2.4 Machine learning modeling and validation

We employed five machine learning methods, namely LR, RF, eXtreme Gradient Boosting (XGBoost), KNN, and support vector machine (SVM), for modeling within the caret framework in R. The data were randomly divided into 10 repetitive groupings based on an 8:2 ratio (training group: validation group) to generate 10 sets of training and validation datasets. We modeled the training sets using the aforementioned five methods.

For all methods except LR, we predefined a wide range of parameters and evaluated them using the Grid Searching method using 3 independent 10-fold cross-validations to obtain the most appropriate modeling parameter within the current parameter space, which was then used for model construction.

We predicted scores on the training set data using the models obtained from the five methods. Based on the prediction results of the training set, we plotted receiver operating characteristic (ROC) curves, and the point closest to the top-left corner was selected as the classification threshold (closest.topleft). We applied the model and threshold to the validation set data and calculated the area under the curve (AUC), sensitivity, and specificity to evaluate the model performance and determine the final resultant model.

### 2.5 Evaluating model performance based on the latest WEO guidelines

We assessed the diagnostic performance of the outcome model in patients with NAA, AA, and CRC in the adenoma-carcinoma sequence using true positive rate (TPR) and false positive rate (FPR). Qualitative FOBT was selected as a validated non-invasive screening test, and we compared the diagnostic performance of the outcome model and the FOBT in patients with FOBT results. We also employed an adjustable positivity threshold to assess disease risk based on an arbitrary risk scale from 0 to 100, calculating the true positives (TP), false negatives (FN), true negatives (TN), false positives (FP), sensitivity (%), specificity (%), positive predictive value (PPV, %), and negative predictive value (NPV, %) (18).

### 2.6 Statistical analysis

To compare the differences among groups, we adopted the Wilcoxon test, t-test, or chi-square test, depending on the type and distribution of the data. We performed ROC curve analysis using the pROC package in R, and the Delong method was used to calculate the confidence intervals. The bootstrap method was applied to calculate the 95% confidence intervals for sensitivity and specificity. The Hanley-McNeil test was used to analyze the statistical significance of the difference in AUC between the outcome model and FOBT (19).

## 3 Results

### 3.1 Study participants

We initially included a total of 575 AAs and 3263 controls. After QC, 569 AAs and 3228 controls were eligible for machine learning modeling and validation. The demographic and clinical characteristics of the study participants are shown in **Table 1**. **Supplementary Table 2** provides more descriptive information on features.

**Table 1:** Demographic and clinical characteristics of the study participants.

Variables	Case n = 569	Control n = 3228	*P*-value
Age, yr, Mean ± SD	61.4 ± 10.2	50.5 ± 13.0	< 0.001
Sex, male, n (%)	369 (64.9)	1208 (37.4)	< 0.001
Weight, kg, Mean ± SD	70.1 ± 11.9	66.2 ± 12.6	< 0.001
Comorbidities, n (%)			
Ischemic cerebrovascular disease	73 (12.8)	301 (9.3)	0.01
Coronary heart disease	90 (15.8)	339 (10.5)	< 0.001
Diabetes mellitus	158 (27.8)	481 (14.9)	< 0.001
Hypertension	235 (41.3)	700 (21.7)	< 0.001

Data are presented as the mean ± SD, median (quartile 1–quartile 3), or N (%).

### 3.2 Feature screening

**Figure 2:**
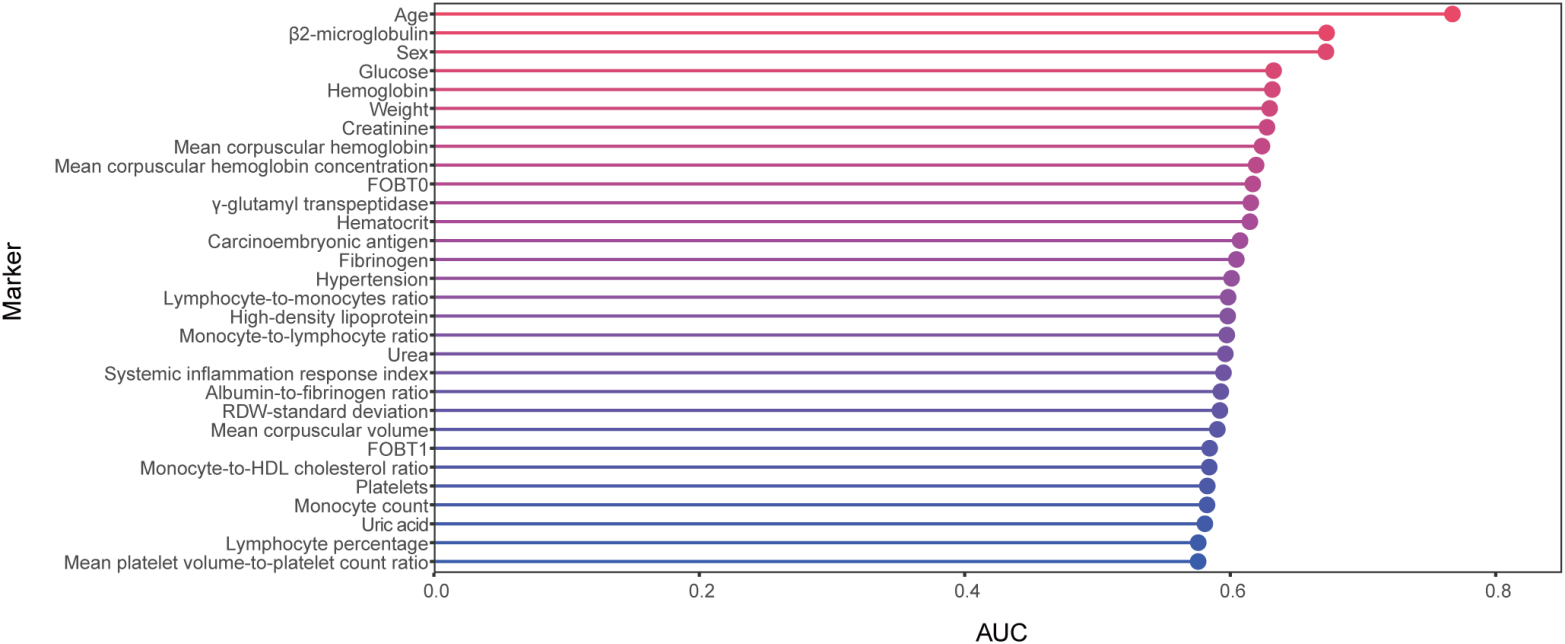
Cleveland dot plots show AUCs for Top 30 parameters identifying AAs and controls. AA, advanced adenoma.

We collected 167 features, and after data QC and feature screening, 60 features were retained for machine learning modeling (see **Supplementary Table 3** for details). In ROC analyses using separate variable to differentiate between the AA and control groups (**Figure 2**, **Table 2**), none of these indicators demonstrated strong discriminatory power (AUC<0.8), with age exhibiting the highest discriminatory power (AUC=0.77).

**Table 2:** Diagnostic performance of various models in training and validation sets.

Methods	AUC	Sensitivity, % (95% CI)	Specificity, % (95% CI)
KNN			
Train	0.938 (0.929-0.946)	90.4 (87.7-93.0)	83.4 (81.9-84.8)
Valid	0.754 (0.708-0.801)	56.6 (47.8-65.5)	78.8 (75.7-81.9)
LR			
Train	0.844 (0.826-0.863)	79.2 (75.2-82.7)	74.8 (73.1-76.4)
Valid	0.833 (0.790-0.875)	77.0 (69.0-84.1)	75.5 (72.1-78.9)
RF			
Train	1	100	100
Valid	0.820 (0.778-0.861)	23.9 (15.9-31.9)	97.2 (95.8-98.5)
SVM			
Train	0.920 (0.904-0.936)	84.7 (81.1-87.9)	91.6 (90.4-92.6)
Valid	0.773 (0.724-0.823)	69.0 (60.2-77.9)	74.6 (71.2-77.8)
XGBoost			
Train	0.955 (0.947-0.963)	87.5 (84.4-90.4)	88.4 (87.2-89.6)
Valid	0.850 (0.813-0.887)	70.8 (62.0-78.8)	83.4 (80.5-86.1)

AUC, area under the curve; CI, confidence interval; LR, logistic regression; RF, random forest; SVM, support vector machine; KNN, k-nearest neighbors; XGBoost, eXtreme Gradient Boosting.

### 3.3 Modelling and validation using different models

**Figure 3:**
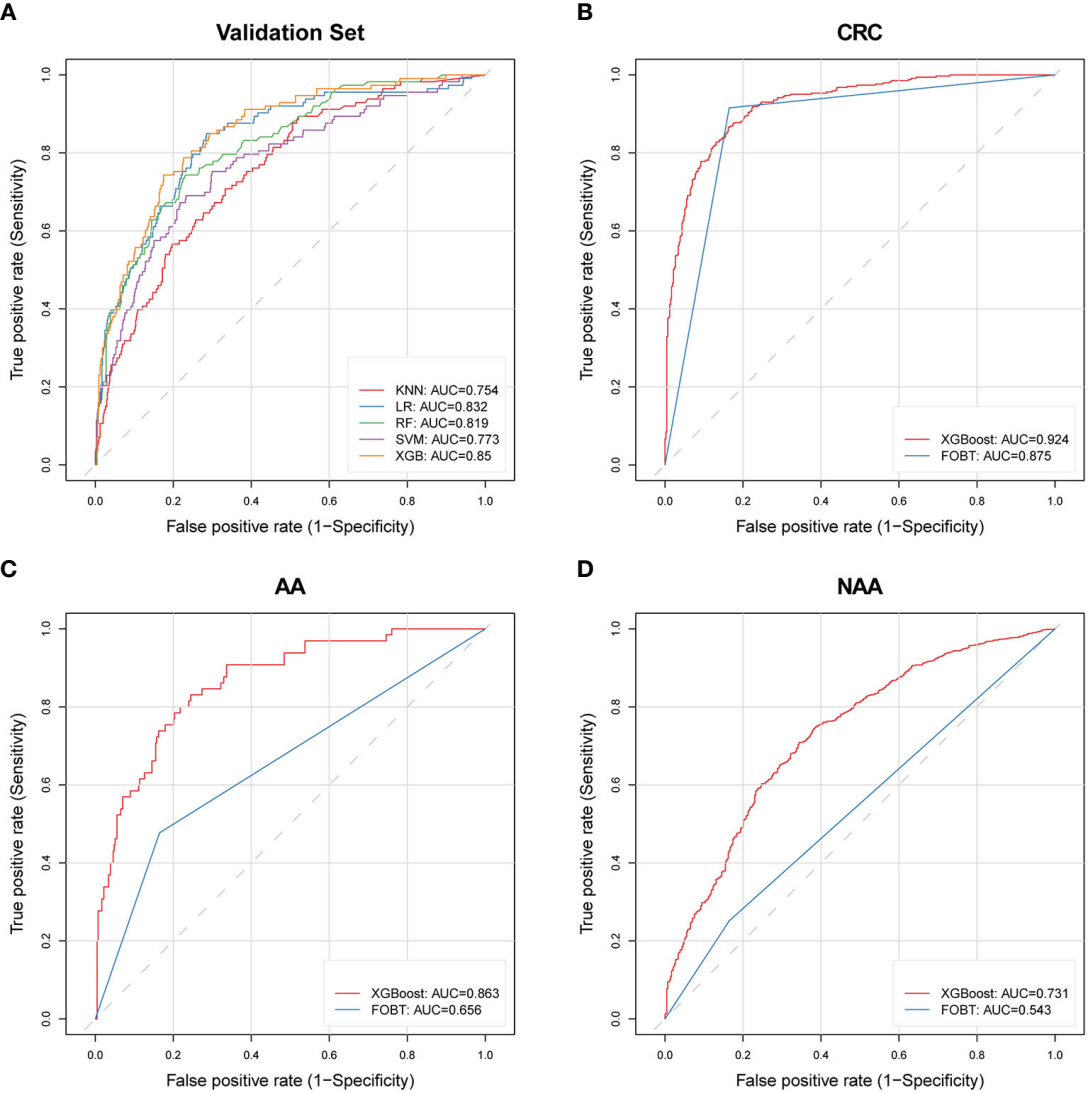
ROC curves of machine learning models and FOBT in different validation cohorts. **(A)** Five constructed machine learning models in the validation set; **(B)** XGBoost model and FOBT in the CRC validation set with FOBT results; **(C)** XGBoost model and FOBT in the AA validation set with FOBT results; **(D)** XGBoost model and FOBT in the NAA validation set that includes FOBT results. AA, advanced adenoma; NAA, non-advanced adenoma; CRC, colorectal cancer; ROC, receiver operating characteristic; XGBoost, eXtreme Gradient Boosting; FOBT, fecal occult blood test; LR, logistic regression; RF, random forest; SVM, support vector machine; KNN, k-nearest neighbors; AUC, area under the curve.

We successfully built five models using different machine learning methods (KNN, XGBoost, LR, RF, and SVM). We calculated the AUC, sensitivity, and specificity for both the training and validation sets to characterize the diagnostic performance of these models (**Figure 3A**, **Table 2**). Overall, the XGBoost model showed the most promising diagnostic performance for identifying patients with AA while maintaining a validation set specificity of at least 0.8. The XGBoost model demonstrated good diagnostic performance in both the training and validation sets, with a sensitivity of 87.5% (95% CI, 84.4−90.4%) (AA=456, Control=2583) and a specificity of 88.4% (95% CI, 87.2−89.6%) in the training set. And the validation set performance resulted in 70.8% (95% CI, 62.0−78.8%) sensitivity and 83.4% (95% CI, 80.5−86.1%) specificity (AA=113, Control=645). Conversely, the RF model, which performed well in the training set, exhibited 97.2% specificity but only 23.9% sensitivity in the validation set, suggesting potential overfitting. The combined diagnostic performance of KNN, LR, and SVM in the validation set did not match that of XGBoost. Thus, based on these results, we concluded that the XGBoost method provided the best performance on the dataset and selected it as the final model.

### 3.4 Evaluating the diagnostic performance of models based on the latest WEO guidelines

#### 3.4.1 Diagnostic performance in the adenoma-carcinoma sequence

We validated the diagnostic performance of the XGBoost model in the validation set for AA (n=113), NAA (n=3047), and CRC (n=488) (**Table 3**). In these three groups, the FPR was 16.6% and the TPR was 70.8% (AA), 42.7% (NAA), and 80.1% (CRC), respectively.

**Table 3:** Diagnostic performance of the XGBoost model and FOBT in the validation set of advanced adenoma, non-advanced adenoma, and colorectal cancer.

Subgroups	Case, n	Control, n	XGBoost				FOBT			
			TP, n	TPR, %	FP, n	FPR, %	TP, n	TPR, %	FP, n	FPR, %
AA	113	645	80	70.8	107	16.6				
With FOBT	65	413	46	70.8	64	15.5	31	47.7	68	16.5
NAA	3047	645	1300	42.7	107	16.6				
With FOBT	1996	413	799	40.0	64	15.5	502	25.2	68	16.5
CRC	488	645	391	80.1	107	16.6				
With FOBT	343	413	291	84.8	64	15.5	314	91.6	68	16.5

AA, advanced adenoma; NAA, non-advanced adenoma; CRC, colorectal cancer; XGBoost, eXtreme Gradient Boosting; FOBT, Fecal occult blood test; TP, true positives; TPR, true positive rate; FP, false positives; FPR, false positive rate.

#### 3.4.2 Comparison of diagnostic performance of XGBoost model and FOBT

In this study, we screened three subsets with FOBT results: CRC (n=343), NAA (n=1996), and AA (n=65). This was done to compare the diagnostic performance of the XGBoost model with that of the FOBT, as shown in **Table 4**. The FPR of the XGBoost model (15.5%) was superior to that of the FOBT (16.5%). The TPR of the XGBoost model for NAA and AA was 40.03% and 70.8%, respectively, which were better than FOBT (25.2% and 47.7%). However, in CRC, the TPR of the XGBoost model (84.8%) was lower than that of FOBT (91.6%). The ROC curves of the XGBoost model and FOBT for these three subsets are depicted in **Figures 3B–D**, respectively. Moreover, we analyzed the difference in AUC between the XGBoost model and FOBT. As shown in **Table 4**, we found that in all three validation sets, the AUC of the XGBoost model was significantly higher than that of FOBT (all *P*<0.05). This indicates that from the perspective of AUC, the XGBoost model outperforms FOBT.

**Table 4:** Comparison of AUC between XGBoost and FOBT in different validation sets.

Validation sets	AUC (95% CI)		Estimate Difference	Z	*P*-value
	XGBoost	FOBT			
AA	0.863 (0.805-0.921)	0.656 (0.580-0.732)	0.207	4.231	< 0.001
NAA	0.731 (0.708-0.755)	0.543 (0.514-0.573)	0.188	9.708	< 0.001
CRC	0.924 (0.903-0.945)	0.875 (0.849-0.902)	0.049	2.865	0.004

#### 3.4.3 Adjustable positivity thresholds for the XGBoost model

We transformed the calculation results of the XGBoost model into risk scores with a score range of 0 to 100, allowing visualization of sensitivity, specificity, and other indicators at different thresholds (**Table 5**). For example, choosing a score of 10 as the positive threshold resulted in a sensitivity of 87.6% and specificity of 64.34%, while a score of 20 yielded a sensitivity of 74.3% and specificity of 82.5.

**Table 5:** Relative risk scores for predicting a diagnosis of AA within the next 30 days at different thresholds in the test set.

Cut off	Negative		Positive		TP, n	FN, n	TN, n	FP, n	Sensitivity, %	Specificity, %	PPV, %	NPV, %
	Total, n (%)	AA, n (%)	Total, n (%)	AA, n (%)								
1	0 (0)	0 (0)	758 (100)	113 (14.91)	113	0	0	645	100	0	14.91	NA
2	52 (6.86)	0 (0)	706 (93.14)	113 (16.01)	113	0	52	593	100	8.06	16.01	100
3	147 (19.39)	2 (1.36)	611 (80.61)	111 (18.17)	111	2	145	500	98.23	22.48	18.17	98.64
4	210 (27.7)	4 (1.9)	548 (72.3)	109 (19.89)	109	4	206	439	96.46	31.94	19.89	98.1
5	258 (34.04)	4 (1.55)	500 (65.96)	109 (21.8)	109	4	254	391	96.46	39.38	21.8	98.45
6	294 (38.79)	6 (2.04)	464 (61.21)	107 (23.06)	107	6	288	357	94.69	44.65	23.06	97.96
7	329 (43.4)	8 (2.43)	429 (56.6)	105 (24.48)	105	8	321	324	92.92	49.77	24.48	97.57
8	378 (49.87)	10 (2.65)	380 (50.13)	103 (27.11)	103	10	368	277	91.15	57.05	27.11	97.35
9	402 (53.03)	10 (2.49)	356 (46.97)	103 (28.93)	103	10	392	253	91.15	60.78	28.93	97.51
10	429 (56.6)	14 (3.26)	329 (43.4)	99 (30.09)	99	14	415	230	87.61	64.34	30.09	96.74
11	451 (59.5)	16 (3.55)	307 (40.5)	97 (31.6)	97	16	435	210	85.84	67.44	31.6	96.45
12	475 (62.66)	18 (3.79)	283 (37.34)	95 (33.57)	95	18	457	188	84.07	70.85	33.57	96.21
13	491 (64.78)	21 (4.28)	267 (35.22)	92 (34.46)	92	21	470	175	81.42	72.87	34.46	95.72
14	502 (66.23)	22 (4.38)	256 (33.77)	91 (35.55)	91	22	480	165	80.53	74.42	35.55	95.62
15	516 (68.07)	24 (4.65)	242 (31.93)	89 (36.78)	89	24	492	153	78.76	76.28	36.78	95.35
16	528 (69.66)	27 (5.11)	230 (30.34)	86 (37.39)	86	27	501	144	76.11	77.67	37.39	94.89
17	533 (70.32)	28 (5.25)	225 (29.68)	85 (37.78)	85	28	505	140	75.22	78.29	37.78	94.75
18	545 (71.9)	29 (5.32)	213 (28.1)	84 (39.44)	84	29	516	129	74.34	80	39.44	94.68
19	552 (72.82)	29 (5.25)	206 (27.18)	84 (40.78)	84	29	523	122	74.34	81.09	40.78	94.75
20	561 (74.01)	29 (5.17)	197 (25.99)	84 (42.64)	84	29	532	113	74.34	82.48	42.64	94.83
25	604 (79.68)	45 (7.45)	154 (20.32)	68 (44.16)	68	45	559	86	60.18	86.67	44.16	92.55
50	718 (94.72)	83 (11.56)	40 (5.28)	30 (75)	30	83	635	10	26.55	98.45	75	88.44
75	758 (100)	113 (14.91)	0 (0)	0 (0)	0	113	645	0	0	100	NA	85.09

AA, advanced adenoma; TP, true positives; FP, false positives; PPV, positive predictive value; NPV, negative predictive value; NA, not records.

## 4 Discussion

The recent guidelines from the WEO for endoscopic CRC screening have incorporated principles such as treating screening as a multistep process, recognizing precursor lesions for CRC as legitimate targets, using FIT as a current comparator, and providing the ability to adjust thresholds for new test positivity. In light of this, we conducted a phase I exploratory study using 60 clinical and laboratory data points to develop and validate an XGBoost model for identifying patients with AA. The model exhibited a sensitivity of 70.8% and specificity of 83.4% in the validation set, successfully detecting NAA with a sensitivity of 42.7% and CRC with a sensitivity of 80.1%. The risk assessment scale, transformed by the XGBoost model, showcased varying levels of disease risk at different positivity thresholds, and notably, the XGBoost model outperformed FOBT in identifying more patients with AA.

Detecting and endoscopically resecting colorectal precancerous lesions, such as AA, has been recognized as an effective method for preventing the occurrence of CRC and reducing CRC-induced mortality (20). Ensuring the identification of AA is a crucial objective in CRC screening programs (21). Despite colonoscopy being the most frequently recommended and performed screening method, its adoption remains low among the Chinese population, with some individuals preferring less invasive alternatives such as FOBT or FIT (22). Additionally, a significant number of subjects show a preference for blood-based screening tests over stool-based tests (23), which poses challenges to the widespread implementation of stool tests. Developing AA identification tools based on easily accessible data without imposing additional burdens on patients or healthcare providers increases the likelihood of enabling patients to benefit from screening. Pan et al. (24) constructed a diagnostic model based on serum N-glycan levels with machine learning involving a population comprising cases of AA and CRC. In this model, the sensitivity and specificity for diagnosing AA were reported as 58% and 85%, respectively. However, it’s important to note that, like many existing CRC diagnostic models, Pan et al. did not create a dedicated model exclusively for AA (25–28). This approach could explain the poor performance of the model in identifying AA. Xiang et al. (20) developed a serum metabolite-based diagnostic model for AA (255 AAs and 178 controls) with a sensitivity of 44.7% and a specificity of 88.9%. The study highlighted that the sensitivity of all current AA diagnostic models remains below 45% at a similar level of specificity. While our model achieves a sensitivity above 50% for detecting AA at this level of specificity, it does not meet the requirements set by certain agencies, such as the United States Preventive Services Task Force, which mandates an acceptable sensitivity of at least 70% for CRC and a specificity of at least 90% for both cancer and advanced precursor lesion (29).

In China, certain large-scale CRC screening programs and hospitals typically employ the qualitative immunogold method for FOBT (30–32). However, there remain individuals who either cannot or choose not to provide stool samples. Importantly, the diagnostic model utilized in this study does not necessarily rely on FOBT results; it is designed to apply to subjects without FOBT. In the current exploratory study of the adenoma-carcinoma sequence, the XGBoost model demonstrated superiority over FOBT in diagnosing NAA and AA but was found to be less effective than FOBT in diagnosing CRC. The simplicity and rapidity of the FOBT, and notably high sensitivity for CRC screening render it irreplaceable in the context of the adenoma-carcinoma sequence. Additionally, the XGBoost model offers potential benefits to patients who do not undergo FOBT for various reasons.

The test positivity threshold plays a crucial role in determining various important parameters. Specifically, it influences the test positivity rate, which subsequently impacts the workload of colonoscopy, the quantity of CRC or AA that warrant detection through colonoscopy (a potentially cost-effective alternative measure), the detection rate of the target lesion, and the positive predictive value (33). Non-invasive screening tests with adjustable positivity thresholds or algorithms enable the selection of test accuracy parameters, including diagnostic sensitivity and specificity, as well as test positivity rates that optimally align with the intended goals of the screening program (5). We present test accuracy parameters at different positivity thresholds in the risk assessment table. The capacity to modify detection thresholds can effectively manage the expenses related to colonoscopy, workforce availability, treatment costs, and the public expectations that are integral to equity-focused programs (5). By offering diverse risk stratification data, it can enhance physicians’ clinical decision-making process for individual patients.

This study has several limitations. Firstly, the study population originates from a single center, warranting further validation in diverse countries or regions to achieve widespread applicability. Secondly, it’s noteworthy that a significant portion of the AA cases were recruited from clinical settings with relatively high prevalence rates, which might limit the full representativeness of our results in the general population. Lastly, certain non-routine laboratory indicators included in this study, such as tumor markers and coagulation function, may contribute to an increase in the cost for the patient.

In conclusion, we adhered to WEO guidelines, constructing the XGBoost model of the AA patients using clinical and laboratory data in a phase I exploratory study. We established adjustable positivity thresholds to accommodate diverse screening program objectives. This model significantly outperforms FOBT in identifying patients with NAA and AA in the adenoma-carcinoma sequence; however, it cannot replace FOBT for CRC patients. Despite the XGBoost model’s substantially improved accuracy in AA identification compared to existing screening methods (e.g., FOBT), it has not yet met the recommendations of certain organizations. Nevertheless, it holds the potential to provide valuable benefits for individuals who are unable to undergo the FOBT test due to various reasons.

## Data availability statement

The raw data supporting the conclusions of this article will be made available by the authors, without undue reservation.

## Ethics statement

The studies involving humans were approved by Medical Ethics Committee of Shijiazhuang Traditional Chinese Medicine Hospital. The studies were conducted in accordance with the local legislation and institutional requirements. The ethics committee/institutional review board waived the requirement of written informed consent for participation from the participants or the participants’ legal guardians/next of kin because the requirements for informed consent were waived due to the retrospective nature.

## Author contributions

HW: Conceptualization, Investigation, Writing – original draft, Writing – review & editing. XC: Conceptualization, Project administration, Writing – original draft. PM: Data curation, Investigation, Writing – original draft. CZ: Data curation, Investigation, Writing – original draft. JL: Data curation, Investigation, Writing – original draft. TPZ: Data curation, Investigation, Writing – original draft. YL: Data curation, Investigation, Writing – original draft. XL: Data curation, Investigation, Writing – original draft. XYS: Data curation, Investigation, Writing – original draft. XXS: Data curation, Investigation, Writing – original draft. TZ: Data curation, Investigation, Writing – original draft. HYZ: Data curation, Investigation, Writing – original draft. ZW: Data curation, Investigation, Writing – original draft. XF: Formal analysis, Methodology, Writing – review & editing. HL: Formal analysis, Methodology, Writing – review & editing. HWZ: Conceptualization, Funding acquisition, Writing – review & editing, Supervision.
