# Supplementary material for: Machine learning-based identification of colorectal advanced adenoma using clinical and laboratory data: a phase I exploratory study in accordance with updated World Endoscopy Organization guidelines for noninvasive colorectal cancer screening tests
Source: Front Oncol. 2024 Feb 23;14:1325514. doi: 10.3389/fonc.2024.1325514 (PMC10921227; doi:10.3389/fonc.2024.1325514)
Supplement: Supplementary file 1 [file Table_1.docx]

Table S1. Laboratory testing methods.

| **Test items** | **Equipment** |
| --- | --- |
| Coagulation function | Automatic coagulation analyzer (Mindray, CX-9000, China) |
| Blood biochemistry | ADVIA Chemistry XPT System (Siemens Healthineers, USA) |
| Blood lipid | ADVIA Chemistry XPT System (Siemens Healthineers, USA) |
| Tumor makers | Automated chemiluminescent immunoassay analyzer (Siemens Healthineers, Atellica IM 1699 Analyzer, USA) |
| Routine blood test | Automatic hematology analyzer (Mindray, BC-7500CS, China) |
| Routine urine test | Automatic urine analysis workstation (Dirui, FUS-2000, China) |
| Fecal occult blood test | Automatic stool extraction system (Orienter, FA180, China) |
