# Supplementary material for: Machine learning-based identification of colorectal advanced adenoma using clinical and laboratory data: a phase I exploratory study in accordance with updated World Endoscopy Organization guidelines for noninvasive colorectal cancer screening tests
Source: Front Oncol. 2024 Feb 23;14:1325514. doi: 10.3389/fonc.2024.1325514 (PMC10921227; doi:10.3389/fonc.2024.1325514)
Supplement: Supplementary file 2 [file Table_2.docx]

**Table S2** All characteristics of participants.

| **Variables** | **Control**  **n = 3228** | **Case**  **n = 569** | ***P*-value** |
| --- | --- | --- | --- |
| Age, year | 51.00 [12.00, 87.00] | 62.00 [25.00, 88.00] | <0.001 |
| Sex, male, n (%) | 1208 (37.4) | 369 (64.9) | <0.001 |
| Weight, kg | 65.00 [35.00, 130.00] | 70.00 [41.00, 118.00] | <0.001 |
| Marital status, n (%) |  |  | <0.001 |
| Married | 2885 (89.4) | 525 (92.3) |  |
| Single | 117 (3.6) | 1 (0.2) |  |
| Divorced | 15 (0.5) | 1 (0.2) |  |
| Widowed | 31 (1.0) | 13 (2.3) |  |
| Others | 180 (5.6) | 29 (5.1) |  |
| Smoking status, n (%) |  |  | <0.001 |
| Never | 2060 (94.9) | 319 (84.4) |  |
| Current | 90 (4.1) | 52 (13.8) |  |
| Former | 21 (1.0) | 7 (1.9) |  |
| Drinking status, n (%) |  |  | <0.001 |
| Never | 2048 (93.6) | 320 (84.0) |  |
| Current | 127 (5.8) | 56 (14.7) |  |
| Former | 12 (0.5) | 5 (1.3) |  |
| Family history of colorectal cancer, n (%) | 42 (1.3) | 10 (1.8) | 0.505 |
| Family history of malignant tumor of digestive system, n (%) | 157 (4.9) | 34 (6.0) | 0.311 |
| Comorbidities, n (%) |  |  |  |
| Hypertension | 700 (21.7) | 235 (41.3) | <0.001 |
| Ischemic cerebrovascular disease | 301 (9.3) | 73 (12.8) | 0.012 |
| Coronary heart disease | 339 (10.5) | 90 (15.8) | <0.001 |
| Hyperlipemia | 301 (9.3) | 59 (10.4) | 0.48 |
| Previous History of cancer | 45 (1.4) | 15 (2.6) | 0.045 |
| Liver diseases | 355 (11.0) | 53 (9.3) | 0.262 |
| Liver cirrhosis | 5 (0.2) | 2 (0.4) | 0.633 |
| Hepatitis | 82 (2.5) | 12 (2.1) | 0.643 |
| Fatty liver | 267 (8.3) | 41 (7.2) | 0.438 |
| Diabetes mellitus | 481 (14.9) | 158 (27.8) | <0.001 |
| Department, n (%) |  |  | 0.001 |
| Department of Integrated Chinese and Western Medicine | 15 (0.5) | 2 (0.4) |  |
| Department of Endocrinology | 135 (4.2) | 26 (4.6) |  |
| Department of Surgery | 167 (5.2) | 31 (5.4) |  |
| Department of Gynecology | 135 (4.2) | 3 (0.5) |  |
| Department of Rehabilitation | 3 (0.1) | 1 (0.2) |  |
| Department of Cardiology | 118 (3.7) | 30 (5.3) |  |
| Department of Dermatology | 29 (0.9) | 7 (1.2) |  |
| Department of Geriatrics | 71 (2.2) | 11 (1.9) |  |
| Department of Anus & Intestine surgery | 1764 (54.6) | 321 (56.4) |  |
| Department of Pulmonology | 20 (0.6) | 5 (0.9) |  |
| Department of Nephropathy | 39 (1.2) | 6 (1.1) |  |
| Department of Oncology | 11 (0.3) | 6 (1.1) |  |
| Department of Vascular | 36 (1.1) | 4 (0.7) |  |
| Department of Neurology | 157 (4.9) | 20 (3.5) |  |
| Department of Gastroenterology | 442 (13.7) | 91 (16.0) |  |
| Department of Acupuncture | 14 (0.4) | 2 (0.4) |  |
| Department of Rheumatology | 14 (0.4) | 0 (0.0) |  |
| Department of Orthopedics | 58 (1.8) | 3 (0.5) |  |
| Routine blood indicators |  |  |  |
| White blood cells, (10^-9^/L) | 5.48 [2.09, 27.03] | 5.84 [2.33, 14.57] | <0.001 |
| Red blood cells, (10^-12^/L) | 4.46 [1.85, 6.31] | 4.58 [1.86, 6.37] | <0.001 |
| Hemoglobin, (%) | 40.90 [14.00, 55.50] | 42.60 [19.80, 54.20] | <0.001 |
| Hemoglobin, (g/L) | 136.00 [43.00, 185.00] | 142.00 [61.00, 181.00] | <0.001 |
| Platelets, (10^-9^/L) | 232.00 [60.00, 755.00] | 216.00 [84.00, 728.00] | <0.001 |
| Platelet-large cell ratio, (%) | 21.40 [3.80, 66.00] | 21.30 [7.50, 52.70] | 0.543 |
| Monocyte percentage, (%) | 5.50 [0.10, 18.80] | 5.60 [0.80, 12.60] | 0.043 |
| Monocyte count, (10^-9^/L) | 0.30 [0.01, 1.71] | 0.33 [0.05, 1.00] | <0.001 |
| Erythrocyte sedimentation rate, (mm/h) | 14.00 [1.00, 136.00] | 13.00 [1.00, 108.00] | 0.391 |
| Red cell distribution width-coefficient of variation, (%) | 12.70 [11.10, 40.50] | 12.80 [11.30, 26.30] | 0.005 |
| Red cell distribution width-standard deviation, (fL) | 41.70 [31.40, 110.80] | 42.50 [34.80, 71.50] | <0.001 |
| Lymphocyte percentage, (%) | 30.00 [2.20, 59.20] | 28.05 [3.40, 56.80] | <0.001 |
| Lymphocyte count, (10^-9^/L) | 1.60 [0.28, 5.33] | 1.59 [0.36, 3.83] | 0.684 |
| Mean corpuscular volume, (fL) | 91.60 [53.10, 134.80] | 92.50 [63.20, 131.00] | <0.001 |
| Mean corpuscular hemoglobin, (pg) | 30.60 [13.30, 45.80] | 30.95 [20.00, 46.50] | <0.001 |
| Mean corpuscular hemoglobin concentration, (g/L) | 332.00 [251.00, 398.00] | 335.00 [297.00, 366.00] | <0.001 |
| Basophil percentage, (%) | 0.40 [0.00, 6.10] | 0.40 [0.00, 5.90] | 0.739 |
| Basophil count, (10^-9^/L) | 0.02 [0.00, 0.28] | 0.02 [0.00, 0.31] | 0.021 |
| Eosinophil percentage, (%) | 1.40 [0.00, 17.30] | 1.50 [0.00, 34.70] | 0.255 |
| Eosinophil count, (10^-9^/L) | 0.08 [0.00, 1.24] | 0.09 [0.00, 2.76] | 0.004 |
| Plateletcrit, (%) | 0.22 [0.07, 0.50] | 0.20 [0.08, 0.61] | <0.001 |
| Platelet distribution width, (%) | 16.00 [7.50, 22.20] | 16.10 [7.80, 17.20] | 0.46 |
| Mean platelet volume, (fL) | 9.40 [6.10, 16.00] | 9.40 [6.80, 14.10] | 0.6 |
| Neutrophil percentage, (%) | 61.80 [30.70, 92.20] | 63.60 [38.30, 91.90] | <0.001 |
| Neutrophil count, (10^-9^/L) | 3.34 [0.84, 21.66] | 3.63 [1.25, 12.93] | <0.001 |
| Biochemical indicators |  |  |  |
| Alanine aminotransferase, (U/L) | 17.00 [1.40, 632.00] | 18.00 [2.00, 703.60] | 0.037 |
| Gamma-glutamyl transpeptidase, (U/L) | 18.00 [6.00, 1, 703.00] | 22.00 [6.00, 853.00] | <0.001 |
| Creatine kinase, (U/L) | 88.00 [14.80, 12, 870.00] | 92.00 [22.00, 596.00] | 0.024 |
| Alkaline phosphatase, (U/L) | 71.00 [17.00, 413.00] | 77.00 [23.00, 252.00] | <0.001 |
| Uric acid, (μmol/L) | 287.00 [64.00, 677.30] | 319.50 [131.00, 674.10] | <0.001 |
| Glucose, (nmol/L) | 5.50 [3.43, 26.40] | 5.88 [1.00, 23.13] | <0.001 |
| Lactate dehydrogenase, (U/L) | 163.15 [62.00, 1, 902.00] | 165.10 [65.00, 403.00] | 0.165 |
| Glycated hemoglobin, (%) | 6.00 [4.60, 13.30] | 6.30 [4.10, 13.80] | 0.205 |
| Aspartate aminotransferase, (U/L) | 19.10 [7.00, 1, 127.00] | 19.10 [9.00, 603.40] | 0.739 |
| Direct bilirubin, (μmol/L) | 2.70 [0.30, 165.00] | 2.90 [0.20, 57.70] | 0.034 |
| Total bile acid, (μmol/L) | 2.50 [0.00, 307.50] | 2.90 [0.20, 100.00] | <0.001 |
| Homocysteine, (mmol/L) | 11.50 [4.90, 86.40] | 13.10 [1.00, 125.60] | <0.001 |
| Serum amylase, (U/L) | 57.30 [17.00, 125.60] | 63.80 [24.30, 178.80] | 0.183 |
| Serum creatinine, (mmol/L) | 60.00 [32.00, 567.00] | 68.00 [34.00, 788.00] | <0.001 |
| Serum urea, (mmol/L) | 4.52 [1.59, 29.98] | 4.90 [1.70, 28.60] | <0.001 |
| Total bilirubin, (μmol/L) | 12.60 [1.30, 229.20] | 12.90 [2.50, 112.30] | 0.557 |
| Total protein, (g/L) | 72.00 [40.00, 88.40] | 71.00 [31.90, 86.50] | <0.001 |
| 5'-Nucleoticlase, (U/L) | 3.00 [0.00, 57.00] | 3.00 [1.00, 39.00] | 0.161 |
| Superoxide dismutase, (U/mL) | 193.00 [99.40, 279.70] | 184.50 [46.80, 239.10] | <0.001 |
| Cholinesterase, (U/L) | 8, 809.00 [2, 072.00, 18, 756.00] | 8, 829.50 [2, 185.00, 15, 085.00] | 0.97 |
| Cystatin C, (mg/L) | 0.79 [0.41, 5.47] | 0.88 [0.56, 6.50] | <0.001 |
| Hydroxybutyrate dehydrogenase, (U/L) | 131.00 [38.00, 984.00] | 130.90 [42.00, 283.00] | 0.354 |
| Prealbumin, (mg/L) | 244.00 [30.20, 496.40] | 256.90 [72.70, 461.30] | 0.001 |
| Albumin, (g/L) | 44.70 [20.00, 57.40] | 44.00 [13.60, 52.70] | <0.001 |
| Globulin, (g/L) | 27.60 [12.40, 48.20] | 26.60 [17.00, 48.80] | <0.001 |
| Albumin/ globulin | 1.60 [0.60, 3.80] | 1.60 [0.70, 2.70] | 0.086 |
| Adenosine deaminase, (U/L) | 8.90 [2.00, 52.10] | 9.00 [4.00, 45.00] | 0.485 |
| β2-microglobulin, (mg/L) | 1.47 [0.50, 30.66] | 1.69 [0.67, 25.71] | <0.001 |
| Serum monoamine oxidase, (U/L) | 4.60 [0.80, 123.00] | 4.80 [0.60, 19.40] | 0.152 |
| Serum myoglobin, (μg/L) | 45.00 [4.00, 729.00] | 51.80 [9.90, 1, 161.00] | <0.001 |
| Nitric oxide, (μmol/L) | 36.20 [6.40, 79.70] | 36.35 [11.50, 77.00] | 0.273 |
| Total cholesterol, (mmol/L) | 4.90 [1.80, 10.42] | 4.74 [1.90, 10.00] | 0.004 |
| Triglyceride, (mmol/L) | 1.30 [0.29, 15.48] | 1.51 [0.40, 13.87] | <0.001 |
| High-density lipoprotein cholesterol, (mmol/L) | 1.34 [0.27, 2.72] | 1.25 [0.21, 2.32] | <0.001 |
| Low-density lipoprotein cholesterol, (mmol/L) | 2.87 [0.09, 6.78] | 2.81 [0.07, 6.69] | 0.009 |
| Apolipoprotein A1, (g/L) | 1.30 [0.30, 2.60] | 1.30 [0.50, 2.10] | 0.009 |
| Apolipoprotein B, (g/L) | 0.90 [0.20, 2.70] | 0.90 [0.40, 2.40] | 0.068 |
| Lipoprotein(a), (mg/L) | 138.00 [0.00, 1, 526.00] | 147.40 [0.00, 1, 487.50] | 0.565 |
| K(+), (mmol/L) | 4.05 [2.59, 5.94] | 4.03 [2.70, 7.37] | 0.259 |
| Cl(-), (mmol/L) | 105.30 [80.00, 114.10] | 105.50 [89.10, 117.10] | 0.14 |
| Na(+) , (mmol/L) | 140.00 [109.50, 150.00] | 140.30 [124.60, 147.10] | 0.001 |
| Ca(2+), (mmol/L) | 2.30 [1.58, 2.79] | 2.29 [1.64, 2.70] | 0.055 |
| Normalized Ca(2+), (mmol/L) | 1.23 [1.05, 1.37] | 1.19 [1.11, 1.38] | 0.006 |
| Mg(2+), (mmol/L) | 0.86 [0.62, 2.57] | 0.87 [0.57, 1.34] | 0.153 |
| Inorganic phosphorus, (mmol/L) | 1.09 [0.26, 2.39] | 1.07 [0.52, 1.85] | 0.066 |
| Ratio indexes |  |  |  |
| Fibrosis-4 | 1.03 [0.11, 21.19] | 1.33 [0.31, 15.08] | <0.001 |
| MPV/ PC, mean platelet volume to platelet count ratio | 0.04 [0.01, 0.21] | 0.04 [0.01, 0.13] | <0.001 |
| PLR, platelet-to-lymphocyte ratio | 145.43 [43.17, 803.19] | 135.61 [38.98, 580.85] | <0.001 |
| SII, systemic immune-inflammation index | 480.35 [92.25, 10, 852.33] | 510.89 [125.61, 5, 611.05] | 0.174 |
| NLR, neutrophil-to-lymphocyte ratio | 2.07 [0.52, 42.56] | 2.28 [0.67, 26.98] | <0.001 |
| MLR, monocyte-to-lymphocyte ratio | 0.18 [0.00, 2.47] | 0.20 [0.03, 1.36] | <0.001 |
| FPR, fibrinogen-to-pre-albumin ratio | 11.07 [3.90, 233.11] | 11.14 [4.55, 113.51] | 0.177 |
| AFR, albumin-to-fibrinogen ratio | 16.32 [4.22, 37.82] | 15.36 [2.59, 27.94] | <0.001 |
| Fibrinogen/ fibrinogen degradation product | 2.04 [0.00, 14.15] | 1.98 [0.02, 283.00] | 0.54 |
| MHR, monocyte-to-high-density lipoprotein ratio | 0.23 [0.01, 2.21] | 0.26 [0.03, 2.38] | <0.001 |
| SIRI, systemic inflammation response index | 0.61 [0.01, 45.11] | 0.73 [0.09, 15.38] | <0.001 |
| LMR, lymphocyte-to-monocyte ratio | 5.44 [0.41, 355.00] | 4.97 [0.74, 32.20] | <0.001 |
| AISI, aggregate index of systemic inflammation | 143.67 [2.41, 11, 503.47] | 157.93 [16.33, 3, 198.30] | 0.001 |
| Urine routine indicators |  |  |  |
| Crystal, n (%) | 26 (5.8) | 18 (12.4) | 0.013 |
| Urine bilirubin, n (%) | 66 (2.2) | 18 (3.5) | 0.11 |
| Urobilinogen, n (%) | 29 (1.0) | 12 (2.3) | 0.016 |
| Urine protein, n (%) | 118 (3.9) | 34 (6.5) | 0.009 |
| Urine glucose, n (%) | 169 (5.6) | 57 (11.0) | <0.001 |
| Urine ketone, n (%) | 150 (5.0) | 17 (3.3) | 0.112 |
| Urine occult blood, n (%) | 905 (30.0) | 144 (27.6) | 0.296 |
| Nitrite, n (%) | 87 (2.9) | 15 (2.9) | 1 |
| Turbidity, n (%) | 38 (3.6) | 6 (2.5) | 0.475 |
| Specific gravity | 1.02 [1.00, 1.05] | 1.01 [1.00, 1.04] | 0.397 |
| Urine conductivity, (mS/cm) | 18.70 [1.30, 38.80] | 17.00 [2.80, 32.50] | 0.028 |
| Calcium oxalate dihydrate crystal, (p/HPF) | 0.00 [0.00, 25.00] | 0.00 [0.00, 13.35] | 0.594 |
| Phosphate crystal, (p/HPF) | 0.00 [0.00, 200.00] | 0.00 [0.00, 0.60] | 0.045 |
| Urine PH | 6.00 [5.00, 9.00] | 6.00 [5.00, 8.50] | 0.974 |
| Urine white blood cells, n (%) | 583 (19.3) | 87 (16.7) | 0.174 |
| Urine white blood cells, (/uL) | 2.60 [0.00, 1, 818.00] | 3.00 [0.00, 11, 321.00] | 0.03 |
| Urine white blood cells (high power field), (p/HPF) | 0.50 [0.00, 569.89] | 0.45 [0.00, 2, 037.80] | 0.22 |
| Urine red blood cells, (/uL) | 3.00 [0.00, 10, 699.00] | 3.00 [0.00, 50, 722.60] | 0.722 |
| Urine red blood cells (high power field), (p/HPF) | 0.30 [0.00, 766.40] | 0.30 [0.00, 9, 130.10] | 0.473 |
| Urine epithelial cell, (/uL) | 3.10 [0.00, 280.90] | 4.30 [0.00, 99.60] | 0.618 |
| Uric acid crystal, (p/HPF) | 0.00 [0.00, 2.35] | 0.00 [0.00, 0.20] | 0.604 |
| Hyaline cast, (/LPF) | 0.00 [0.00, 3.00] | 0.00 [0.00, 15.00] | 0.001 |
| Calcium oxalate monohydrate crystal, (p/HPF) | 0.00 [0.00, 54.80] | 0.00 [0.00, 13.85] | 0.885 |
| Coagulation function indicators |  |  |  |
| D-dimer, (mg/L) | 0.19 [0.00, 7.95] | 0.23 [0.00, 33.60] | <0.001 |
| Activated partial thromboplastin time, (s) | 28.20 [18.20, 63.30] | 27.40 [17.50, 93.60] | 0.059 |
| Antithrombin III, (%) | 101.55 [38.60, 146.00] | 99.10 [52.20, 139.30] | 0.05 |
| Thrombin time, (s) | 16.20 [10.40, 24.50] | 16.10 [12.10, 21.00] | 0.11 |
| Prothrombin time-international normalized ratio | 0.99 [0.70, 2.40] | 0.99 [0.81, 1.59] | 0.772 |
| Prothrombin time, (s) | 11.90 [9.30, 27.20] | 11.90 [9.70, 19.00] | 0.522 |
| Fibrin degradation product, (mg/L) | 1.30 [0.00, 26.97] | 1.42 [0.01, 125.00] | 0.02 |
| Fibrinogen, (g/L) | 2.73 [1.19, 9.47] | 2.88 [1.70, 11.51] | <0.001 |
| Thyroid function indicators |  |  |  |
| Thyroid stimulating hormone, (mIU/L) | 1.90 [0.00, 150.00] | 1.72 [0.33, 8.90] | 0.133 |
| Thyroxine, (nmol/L) | 105.00 [51.75, 221.20] | 98.50 [56.74, 159.80] | 0.164 |
| Triiodothyronine, (nmol/L) | 1.66 [0.84, 8.68] | 1.67 [0.81, 3.12] | 0.249 |
| Free thyroxine, (pmol/L) | 15.79 [3.02, 40.42] | 15.96 [11.04, 25.12] | 0.019 |
| Free triiodothyronine, (pmol/L) | 4.70 [2.48, 26.69] | 4.76 [1.80, 7.04] | 0.661 |
| Antithyroid peroxidase autoantibody, (U/mL) | 28.00 [3.60, 3, 841.20] | 28.00 [5.00, 3, 418.60] | 0.017 |
| Tumor marker indicators |  |  |  |
| Carcinoembryonic antigen, (μg/L) | 1.10 [0.00, 16.50] | 1.68 [0.10, 29.32] | <0.001 |
| Alpha fetoprotein, (μg/L) | 2.70 [0.10, 168.90] | 3.00 [0.10, 22.50] | 0.002 |
| Total prostate specific antigen, (μg/L) | 0.78 [0.01, 22.92] | 1.02 [0.11, 13.56] | <0.001 |
| Carbohydrate antigen 125, (U/mL) | 7.40 [0.16, 709.50] | 7.00 [1.20, 1, 000.00] | 0.054 |
| Carbohydrate antigen 153, (U/mL) | 7.24 [0.10, 127.10] | 8.14 [0.50, 50.30] | 0.006 |
| Carbohydrate antigen 199, (U/mL) | 11.37 [0.00, 1, 824.60] | 11.82 [0.01, 174.00] | 0.695 |
| Carbohydrate antigen 724, (U/mL) | 1.82 [0.00, 822.00] | 1.42 [0.20, 161.00] | 0.043 |
| Arterial blood gas analysis |  |  |  |
| Partial pressure of carbon dioxide, (mmHg) | 39.85 [18.60, 56.50] | 38.95 [30.60, 47.30] | 0.766 |
| Carbon dioxide combining power, (mmol/L) | 25.20 [7.00, 36.30] | 25.60 [13.10, 35.50] | 0.082 |
| Base excess, (mmol/L) | 0.00 [-22.00, 4.00] | 1.00 [0.00, 7.00] | 0.077 |
| Lactic acid, (mmol/L) | 0.96 [0.44, 2.21] | 0.78 [0.37, 2.40] | 0.222 |
| PH | 7.40 [7.14, 7.65] | 7.38 [7.31, 7.47] | 0.231 |
| Bicarbonate, (mmol/L) | 24.45 [6.80, 30.70] | 24.50 [17.20, 30.60] | 0.52 |
| Fecal routine indicators |  |  |  |
| Fecal white blood cell, n (%) | 12 (0.5) | 5 (1.2) | 0.169 |
| Fecal red blood cell, n (%) | 31 (1.3) | 14 (3.4) | 0.003 |
| Fecal color, n (%) |  |  | 0.495 |
| Red | 16 (0.7) | 5 (1.2) |  |
| Yellow | 2337 (99.1) | 400 (98.5) |  |
| Black | 6 (0.3) | 1 (0.2) |  |
| Fecal property, n (%) |  |  | 0.368 |
| Soft | 2056 (87.2) | 344 (84.7) |  |
| Hard | 6 (0.3) | 0 (0.0) |  |
| Loose | 291 (12.3) | 62 (15.3) |  |
| Mucous | 3 (0.1) | 0 (0.0) |  |
| Bloody | 1 (0.0) | 0 (0.0) |  |
| Fecal transferrin, n (%) | 363 (25.6) | 111 (43.5) | <0.001 |
| Mold cells, n (%) | 19 (0.8) | 1 (0.2) | 0.362 |
| Fecal occult blood test, n (%) | 369 (17.0) | 184 (52.3) | <0.001 |
| Fat globules, n (%) | 8 (0.4) | 3 (0.8) | 0.548 |
| Blood type, n (%) |  |  | 0.205 |
| A | 387 (23.4) | 81 (26.6) |  |
| AB | 187 (11.3) | 25 (8.2) |  |
| B | 531 (32.2) | 106 (34.9) |  |
| O | 546 (33.1) | 92 (30.3) |  |
| N-terminal B-type natriuretic peptide precursor, (pg/mL) | 75.80 [7.20, 35, 000.00] | 190.00 [2.00, 4, 010.00] | 0.006 |
| Amyloid A, (mg/L) | 5.00 [0.05, 204.86] | 5.00 [1.62, 144.22] | 0.988 |
| Serum procalcitonin, (ng/mL) | 0.05 [0.00, 11.85] | 0.05 [0.00, 3.00] | 0.42 |
| Treponema pallidum antibody, (s/co) | 0.03 [0.00, 360.31] | 0.02 [0.00, 270.44] | 0.8 |
| Cardiac troponin I, (μg/L) | 0.01 [0.01, 0.61] | 0.01 [0.01, 0.40] | 0.51 |
| High-sensitivity C-reactive protein, (mg/L) | 1.40 [0.00, 214.40] | 2.13 [0.00, 161.80] | <0.001 |

Data are presented as the median (quartile 1–quartile 3), or N (%).
