# Supplementary material for: Machine learning-based identification of colorectal advanced adenoma using clinical and laboratory data: a phase I exploratory study in accordance with updated World Endoscopy Organization guidelines for noninvasive colorectal cancer screening tests
Source: Front Oncol. 2024 Feb 23;14:1325514. doi: 10.3389/fonc.2024.1325514 (PMC10921227; doi:10.3389/fonc.2024.1325514)
Supplement: Supplementary file 3 [file Table_3.docx]

**Table S3** Characteristics of participants included in the outcome model.

| **Variables** | **Control**  **n = 3228** | **Case**  **n = 569** | ***P*-value** |
| --- | --- | --- | --- |
| Age, year | 51.00 [12.00, 87.00] | 62.00 [25.00, 88.00] | <0.001 |
| Sex, male, n (%) | 1208 (37.4) | 369 (64.9) | <0.001 |
| Weight, kg | 65.00 [35.00, 130.00] | 70.00 [41.00, 118.00] | <0.001 |
| Comorbidities, n (%) |  |  |  |
| Hypertension | 700 (21.7) | 235 (41.3) | <0.001 |
| Ischemic cerebrovascular disease | 301 (9.3) | 73 (12.8) | 0.012 |
| Coronary heart disease | 339 (10.5) | 90 (15.8) | <0.001 |
| Diabetes mellitus | 481(14.9) | 158 (27.8) | <0.001 |
| Routine blood indicators |  |  |  |
| White blood cells, (10^-9^/L) | 5.48 [2.09, 27.03] | 5.84 [2.33, 14.57] | <0.001 |
| Red blood cells, (10^-12^/L) | 4.46 [1.85, 6.31] | 4.58 [1.86, 6.37] | <0.001 |
| Hematocrit, (%) | 40.90 [14.00, 55.50] | 42.60 [19.80, 54.20] | <0.001 |
| Hemoglobin, (%) | 136.00 [43.00, 185.00] | 142.00 [61.00, 181.00] | <0.001 |
| Platelets, (10^-9^/L) | 232.00 [60.00, 755.00] | 216.00 [84.00, 728.00] | <0.001 |
| Platelet-large cell ratio, (%) | 21.40 [3.80, 66.00] | 21.30 [7.50, 52.70] | 0.543 |
| Monocyte count, (10^-9^/L) | 0.30 [0.01, 1.71] | 0.33 [0.05, 1.00] | <0.001 |
| Red cell distribution width-coefficient of variation, (%) | 12.70 [11.10, 40.50] | 12.80 [11.30, 26.30] | 0.005 |
| Red cell distribution width-standard deviation, (fL) | 41.70 [31.40, 110.80] | 42.50 [34.80, 71.50] | <0.001 |
| Lymphocyte percentage, (%) | 30.00 [2.20, 59.20] | 28.05 [3.40, 56.80] | <0.001 |
| Lymphocyte count, (10^-9^/L) | 1.60 [0.28, 5.33] | 1.59 [0.36, 3.83] | 0.684 |
| Mean corpuscular volume, (fL) | 91.60 [53.10, 134.80] | 92.50 [63.20, 131.00] | <0.001 |
| Mean corpuscular hemoglobin, (pg) | 30.60 [13.30, 45.80] | 30.95 [20.00, 46.50] | <0.001 |
| Mean corpuscular hemoglobin concentration, (g/L) | 332.00 [251.00, 398.00] | 335.00 [297.00, 366.00] | <0.001 |
| Neutrophil percentage, (%) | 61.80 [30.70, 92.20] | 63.60 [38.30, 91.90] | <0.001 |
| Neutrophil count, (10^-9^/L) | 3.34 [0.84, 21.66] | 3.63 [1.25, 12.93] | <0.001 |
| Biochemical indicators |  |  |  |
| Albumin, (g/L) | 44.70 [20.00, 57.40] | 44.00 [13.60, 52.70] | <0.001 |
| Alanine aminotransferase, (U/L) | 17.00 [1.40, 632.00] | 18.00 [2.00, 703.60] | 0.037 |
| Gamma-glutamyl transpeptidase, (U/L) | 18.00 [6.00, 1, 703.00] | 22.00 [6.00, 853.00] | <0.001 |
| Alkaline phosphatase, (U/L) | 71.00 [17.00, 413.00] | 77.00 [23.00, 252.00] | <0.001 |
| Uric acid, (μmol/L) | 287.00 [64.00, 677.30] | 319.50 [131.00, 674.10] | <0.001 |
| Glucose, (nmol/L) | 5.50 [3.43, 26.40] | 5.88 [1.00, 23.13] | <0.001 |
| Aspartate aminotransferase, (U/L) | 19.10 [7.00, 1, 127.00] | 19.10 [9.00, 603.40] | 0.739 |
| Serum creatinine, (mmol/L) | 60.00 [32.00, 567.00] | 68.00 [34.00, 788.00] | <0.001 |
| Serum urea, (mmol/L) | 4.52 [1.59, 29.98] | 4.90 [1.70, 28.60] | <0.001 |
| Total cholesterol, (mmol/L) | 4.90 [1.80, 10.42] | 4.74 [1.90, 10.00] | 0.004 |
| Triglyceride, (mmol/L) | 1.30 [0.29, 15.48] | 1.51 [0.40, 13.87] | <0.001 |
| Low-density lipoprotein cholesterol, (mmol/L) | 2.87 [0.09, 6.78] | 2.81 [0.07, 6.69] | 0.009 |
| High-density lipoprotein cholestero, (mmol/L) | 1.34 [0.27, 2.72] | 1.25 [0.21, 2.32] | <0.001 |
| Total bilirubin, (μmol/L) | 12.60 [1.30, 229.20] | 12.90 [2.50, 112.30] | 0.557 |
| Total protein, (g/L) | 72.00 [40.00, 88.40] | 71.00 [31.90, 86.50] | <0.001 |
| Cholinesterase, (U/L) | 8, 809.00 [2, 072.00, 18, 756.00] | 8, 829.50 [2, 185.00, 15, 085.00] | 0.97 |
| β2-microglobulin, (mg/L) | 1.47 [0.50, 30.66] | 1.69 [0.67, 25.71] | <0.001 |
| Lipoprotein(a), (mg/L) | 138.00 [0.00, 1, 526.00] | 147.40 [0.00, 1, 487.50] | 0.565 |
| Total bile acid, (μmol/L) | 2.50 [0.00, 307.50] | 2.90 [0.20, 100.00] | <0.001 |
| Ratio indexes |  |  |  |
| MPV/ PC, mean platelet volume to platelet count ratio | 0.04 [0.01, 0.21] | 0.04 [0.01, 0.13] | <0.001 |
| PLR, platelet-to-lymphocyte ratio | 145.43 [43.17, 803.19] | 135.61 [38.98, 580.85] | <0.001 |
| NLR, neutrophil-to-lymphocyte ratio | 2.07 [0.52, 42.56] | 2.28 [0.67, 26.98] | <0.001 |
| MLR, monocyte-to-lymphocyte ratio | 0.18 [0.00, 2.47] | 0.20 [0.03, 1.36] | <0.001 |
| AFR, albumin-to-fibrinogen ratio | 16.32 [4.22, 37.82] | 15.36 [2.59, 27.94] | <0.001 |
| MHR, monocyte-to-high-density lipoprotein ratio | 0.23 [0.01, 2.21] | 0.26 [0.03, 2.38] | <0.001 |
| SIRI, systemic inflammation response index | 0.61 [0.01, 45.11] | 0.73 [0.09, 15.38] | <0.001 |
| LMR, lymphocyte-to-monocyte ratio | 5.44 [0.41, 355.00] | 4.97 [0.74, 32.20] | <0.001 |
| Urine routine indicators |  |  |  |
| Urine glucose, n (%) | 169 (5.6) | 57 (11.0) | <0.001 |
| Coagulation function indicators |  |  |  |
| D-dimer, (mg/L) | 0.19 [0.00, 7.95] | 0.23 [0.00, 33.60] | <0.001 |
| Activated partial thromboplastin time, (s) | 28.20 [18.20, 63.30] | 27.40 [17.50, 93.60] | 0.059 |
| Prothrombin time, (s) | 11.90 [9.30, 27.20] | 11.90 [9.70, 19.00] | 0.522 |
| Fibrinogen, (g/L) | 2.73 [1.19, 9.47] | 2.88 [1.70, 11.51] | <0.001 |
| Tumor marker indicators |  |  |  |
| Carcinoembryonic antigen, (μg/L) | 1.10 [0.00, 16.50] | 1.68 [0.10, 29.32] | <0.001 |
| Alpha fetoprotein, (μg/L) | 2.70 [0.10, 168.90] | 3.00 [0.10, 22.50] | 0.002 |
| Carbohydrate antigen 125, (U/mL) | 7.40 [0.16, 709.50] | 7.00 [1.20, 1, 000.00] | 0.054 |
| Carbohydrate antigen 199, (U/mL) | 11.37 [0.00, 1, 824.60] | 11.82 [0.01, 174.00] | 0.695 |
| Stool routine indicators |  |  |  |
| Fecal occult blood test, n (%) | 369 (17.0) | 184 (52.3) | <0.001 |

Data are presented as the median (quartile 1–quartile 3), or N (%).
